# *Spermatogenesis Associated 4* Promotes Sertoli Cell Proliferation Modulated Negatively by Regulatory Factor X1

**DOI:** 10.1371/journal.pone.0075933

**Published:** 2013-10-11

**Authors:** Junjun Jiang, Nannan Zhang, Hiroshi Shiba, Liyuan Li, Zhao Wang

**Affiliations:** 1 MOE Key Laboratory of Protein Sciences, Department of Pharmacology, School of Medicine, Tsinghua University, Beijing, China; 2 Department of Bioinformatics, Graduate School of Bioscience and Biotechnology, Tokyo Institute of Technology, Tokyo, Japan; Ecole Normale Superieure de Lyon, France

## Abstract

Spermatogenesis associated 4 (*Spata4*), a testis-specific and CpG island associated gene, is involved in regulating cell proliferation, differentiation and apoptosis. To obtain insight into the role of *Spata4* in cell cycling control, we characterized the promoter region of *Spata4* and investigated its transcriptional regulation mechanism. The *Spata4* promoter is unidirectional transcribed and possesses multiple transcription start sites. Moreover, we present evidence that regulatory factor X1 (RFX1) could bind the typical 14-bp *cis*-elements of *Spata4* promoter, modulate transcriptional activity and endogenous expression of *Spata4*, and further regulate the proliferation of Sertoli cells. Overexpression of RFX1 was shown to down-regulate both the promoter activity and mRNA expression of Spata4, whereas knockdown of RFX1 demonstrated the opposite effects. Our studies provide insight into *Spata4* gene regulation and imply the potential role of RFX1 in growth of Sertoli cells. RFX1 may have negative effect on cell proliferation of Sertoli cells via modulating *Spata4* expression levels by binding the conserved 14-bp *cis*-elements of *Spata4* promoter.

## Introduction

Spermatogenesis associated 4 (*Spata4*) was initially identified in human testes and plays an important role in cryptorchidism development [1]. All of human, mouse and rat *Spata4* genes have specifically high expression in testis, and among numerous genes that expressed in testis, only minority are testis specific genes [2]–[5]. Therefore, it was proposed that Spata4 participates in the spermatogenesis process [1], and may function during adolescence and maintain spermatogenesis ability, as transcript expression profiles indicated [1], [6]. In addition, recent studies indicate that Spata4 modulates cell growth, proliferation and differentiation in various cell types [1], [7], [8]. Sertoli cells produce essential factors for germ cell development and modulate male fertility [9]. And these factors are of great importance in the process of spermatogenesis [10], [11]. Clearly, the number and function of Sertoli cells determine testicular size, germ cell numbers and spermatozoa output [12]. Thus, Sertoli cell growth and proliferation may contribute to maintaining the normal physiological functions of male reproductive system.

CpG islands are often linked to promoter regions of genes [13]. The accepted definition of CpG island is regions of DNA longer than 200 bp, with a guanine/cytosine content above 50% and an ratio of observed to expected CpG presence above 0.6 [14]. The human, mouse and rat *Spata4* genes showed excellent similarity of the promoter regions, especially a highly conserved region around the start codon between human and mouse *Spata4* genes [6], and the CpG islands of these species also have a high similarity with each other, suggesting that *Spata4* genes of these species may have some common transcription factors.

The RFX (regulatory factor for X-box) family of transcription factors consists of seven members in mammals and shares a highly conserved DNA-binding domain (DBD) [15], [16]. Besides the central located DBD domain, a conserved C-terminal region exists in RFX1 protein [17], mediating the dimerization and transcriptional repression of winged-helix RFX1 protein. RFX1 is involved in transcriptional regulation of a variety of viral and cellular genes, possesses both transcription activation and repression properties [18]. RFX1 was implicated in positive regulation of transcription from enhancer of hepatitis virus (*HBV*) [17] and MHC class II genes [19], [20], and negative effect was observed in transcriptional regulation of cellular genes including *c-myc* [21], [22], *collagen α2* [23], [24], proliferating cell nuclear antigen (*PCNA*) [25], inhibitor of DNA binding 2 (*Id2*) [26], and fibroblast growth factor 1 (*FGF1*) [27]–[29]. Nevertheless, the regulatory targets of RFX1 are still not fully understood. RFX1 is expressed in various tissues. Moreover, RFX1 is prominent both in the somatic Sertoli cells of seminiferous tubules and also detected in early haploid germ cells [30], which suggested a possible role of RFX1 in male reproductive system.

Although the function of *Spata4* is partially uncovered, the transcriptional regulatory mechanism of *Spata4* remains to be clarified. In this study, we presented evidence that expression of *Spata4* could be modulated by RFX1, which binds to the conserved 14-bp *cis*-elements of *Spata4* promoter region. Overexpression of RFX1 could down-regulate *Spata4* expression, whereas RNAi knockdown of RFX1 demonstrated the opposite effects. Moreover, RFX1 is involved in Spata4 mediated Sertoli cell growth. Our findings provide insights into *Spata4* gene regulation and suggest the possible role of the RFX1, the negative transcriptional regulator of *spata4*, in Sertoli cell growth.

## Results

### Identification and characterization of the mouse *Spata4* promoter region

**Figure 1:**
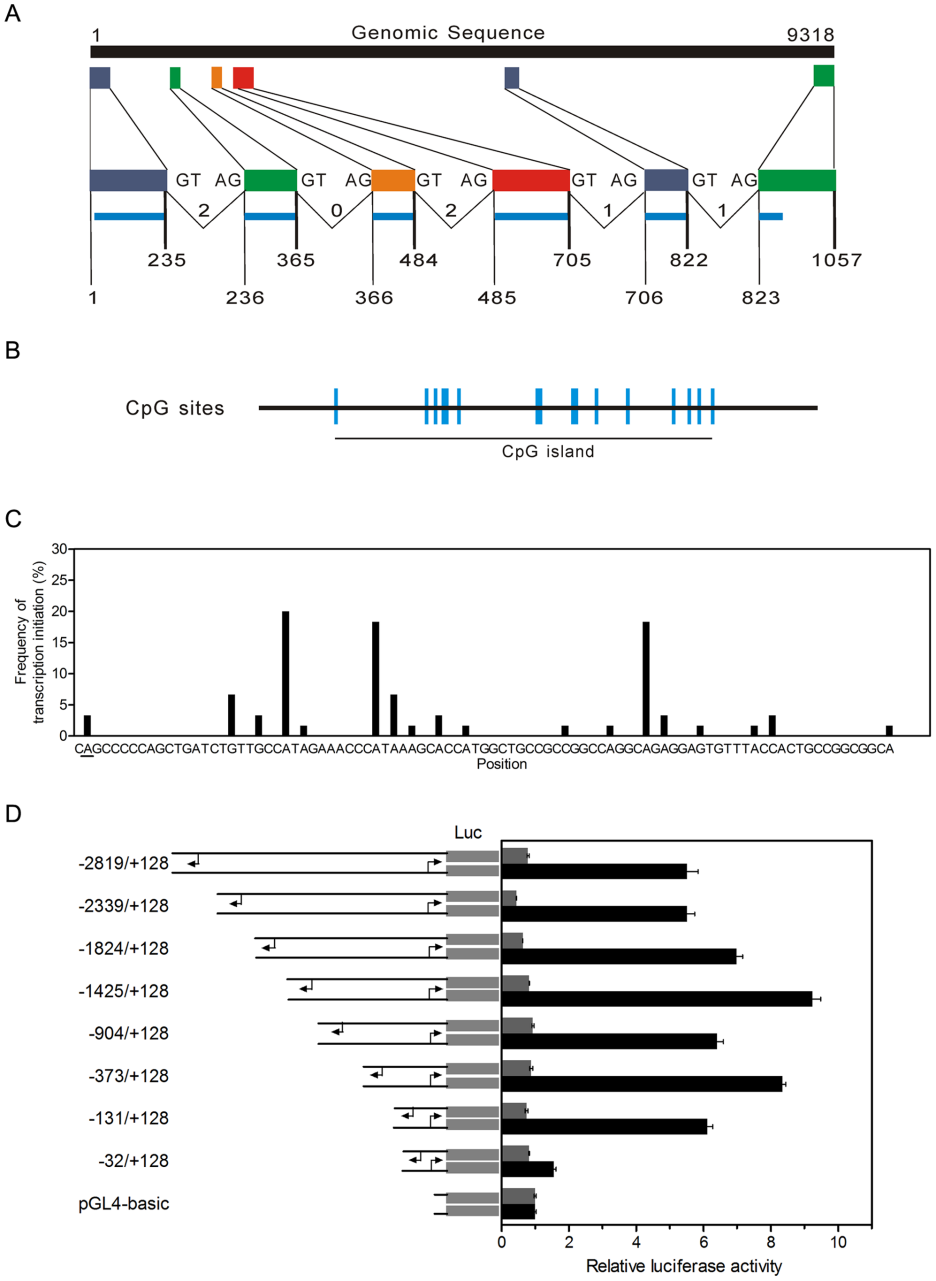
Structure of the mouse *Spata4* gene and identification of the promoter region. (**A**) Exon-intron structure of the *Spata4* gene (1–9318) and mRNA (1–1057) as deduced by MGAlignIt using accession numbers NC_000074.5 and NM_133711.3, respectively. The colored boxes indicate the different exons. The top series of gray colored bars corresponds to individual exons drawn according to their size and position within the genomic sequence. Intron phases (0, 1 or 2) and exon borders are indicated on the bottom series of blue colored bars. (**B**) Mouse *Spata4* promoter contains a CpG island with a length of approximately 400 bp. (**C**) The transcription start sites of mouse *Spata4* gene. The frequency of transcription initiation from different sites is shown. The *Spata4* promoter contains dispersed transcription start sites located within a 90 bp region. The first transcription start site is underlined. (**D**) Reporter activity assay of serially deleted promoter constructs containing different lengths of the 5′-flanking sequence of mouse *Spata4* gene. The plasmids were transiently transfected into TM4 cells, and the reporter activity was analyzed 18 h after transfection. Gray bar, reverse promoter sequences; Black bar, forward promoter sequences. The values are shown as relative ratio to that of pGL4.17 control vector.

The mouse *Spata4* gene locates on chromosome 8 B3.1 and consists of 6 exons (Figure 1A). The mouse *Spata4* promoter contains a CpG island approximately 400-bp-long (Figure 1B) with GC %  = 54% and ObsCpG/ExpCpG  = 0.604, which comply with the accepted definition of CpG island [14]. To identify the promoter region of mouse *Spata4*, we first determined its transcription start site using the 5′-rapid amplification of cDNA ends method (5′-RACE). The *Spata4* promoter contains dispersed transcription start sites located within a 90 bp region (Figure 1C). Transcription of *Spata4* initiates with a purine at +1 at a frequency of 98% and with a pyrimidine-purine dinucleotide at position −1, +1 at a frequency of 88%, which is consistent with previous studies that RNA polymerase II-mediated transcription initiation in mammals is preferentially initiated at a pyrimidine-purine dinucleotide at position −1, +1 [31].

Searching the mouse *Spata4* promoter showed no TATA box, initiator (Inr) or other typical core promoter elements around the transcription start sites. To determine the region that is essential for *Spata4* transcription, we serially truncated the 5′-flanking region of the *Spata4* promoter and analyzed its transcriptional activity through luciferase reporter assays. Deletion of the 5′-flanking region of the *Spata4* promoter from −2819 to −131 retained its transcriptional activity, while further deletion to −32 abolished its transcriptional activity. Therefore, the region between −131 and +128 showed basal transcriptional activity (Figure 1D). Besides, the *Spata4* promoter constructs showed little reverse transcription activity, indicating that the *Spata4* promoter is unidirectional transcribed (Figure 1D).

### *Spata4* promoter contains a functional RFX1 binding site

**Figure 2:**
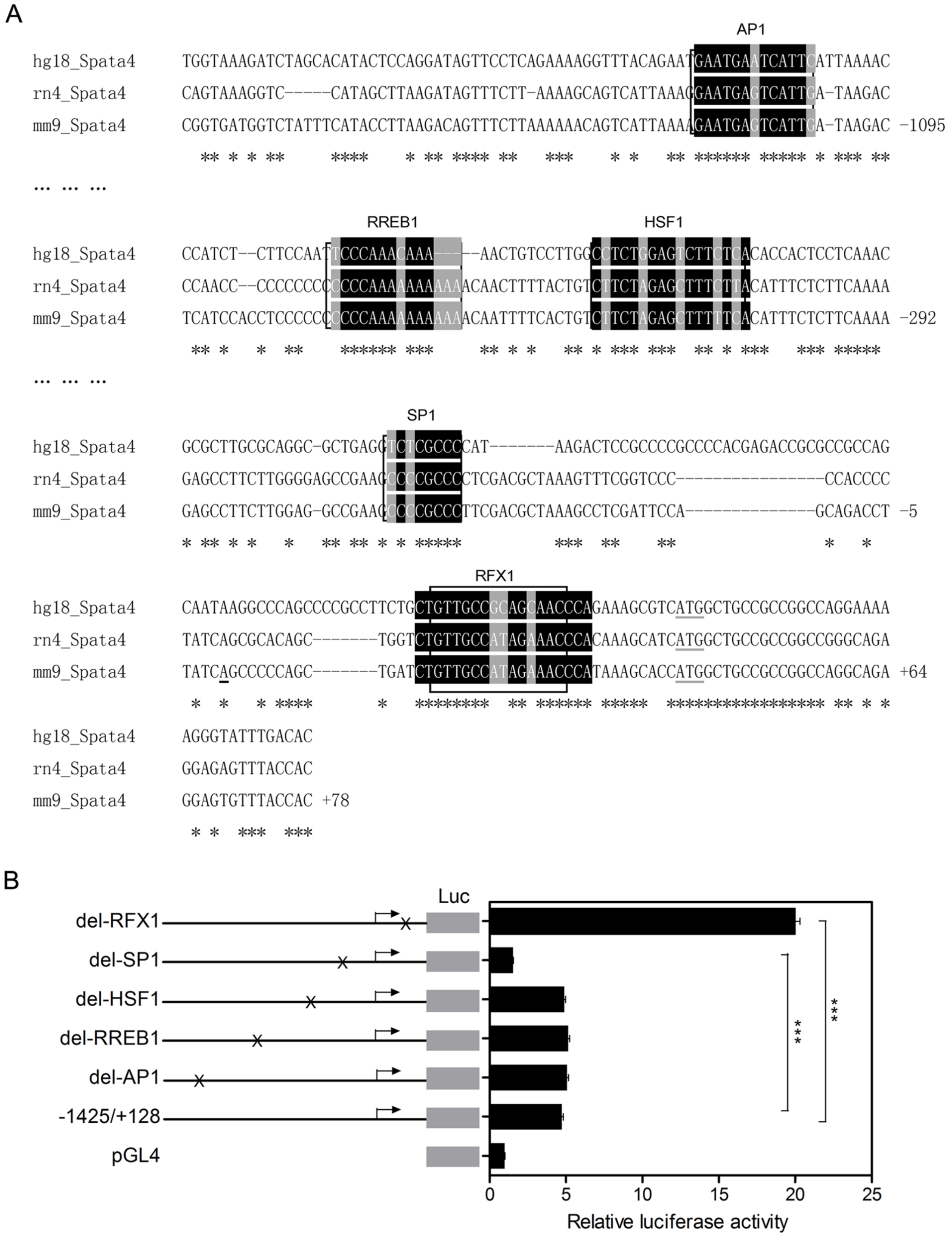
The Spata4 promoter contains a functional RFX1 binding site. (**A**) Nucleotide sequences of the 5′-flanking region of the Mm/hg/rn *Spata4* genes. The 5′-flanking sequences are numbered with respect to the transcription start site, Mulan combined with multiTF (http://multitf.dcode.org/) scanning revealed several conserved potential binding sites of different transcription factors. (**B**) Effect of deletion mutants on the relative luciferase activity of the *Spata4* promoter region. TM4 cells were co-transfected with the −1425/+128 *Spata4* promoter construct and each deletion mutant. The results are the means ± S.E.M. of three independent experiments performed in triplicate. ***P<0.001.

Bioinformatics analysis through combination of Mulan and multi-TF scanning [32], [33] showed that the *Spata4* promoter contains conserved binging sites of several transcription factors, including c-jun (key component of AP-1 complex), RREB1, HSF1, SP1 and RFX1 (Figure 2A). To address which factor could regulate *Spata4* promoter activity, we serially deleted their binding sites from the −1425/+128 reporter construct. Deletion of the RFX1 binding site resulted in 4.2-fold increase of luciferase activity, while deletion of SP1 binding site decreased the luciferase activity by 0.67-fold. Deletion of AP1, RREB1 and HSF1 binding sites had little effect (Figure 2B), compared to the luciferase activity of −1425/+128 construct, respectively. These results indicate that RFX1 is a negative modulator of mouse *Spata4* promoter and SP1 binding sites which locate between −131, −32 might be essential for the basal transcriptional activity of *Spata4*.

**Figure 3:**
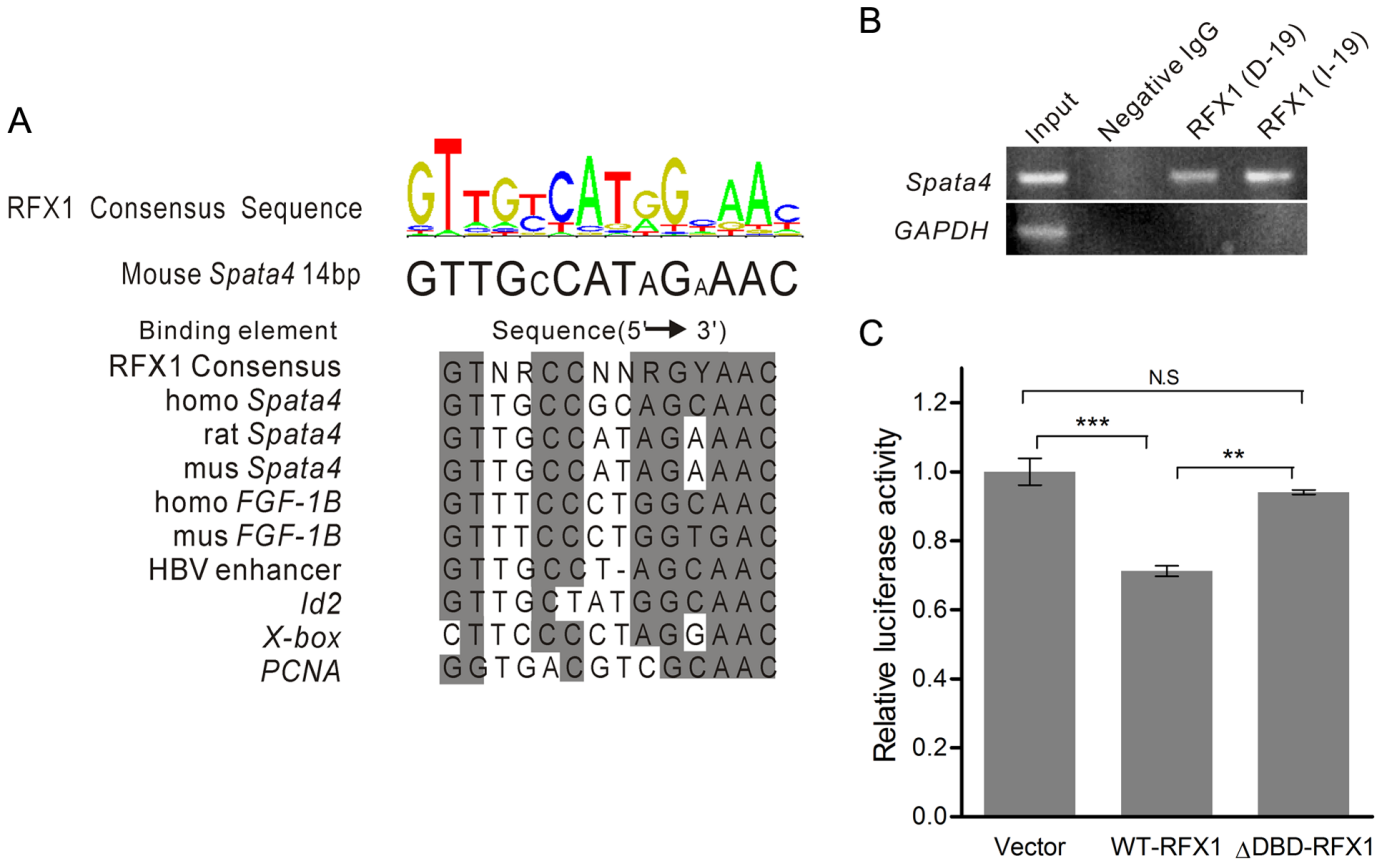
RFX1 binds to the *Spata4* promoter through its DNA binding domain. **(A)** Alignment of the conserved 14-bp sequences of human, rat and mouse *Spata4* promoter with other RFX1 binding sequences shows high similarity to the RFX1 consensus binding sites. (**B**) Chromatin immunoprecipitation assay confirm that RFX1 could bind the conserved 14-bp sequence in mouse Sertoli cells. (**C**) DNA binding domain is essential for the binding of RFX1 to the conserved sequence of *Spata4* promoter. Vector, pcDNA3.1 plasmid; WT-RFX1, pcDNA3.1-RFX1 plasmid; ΔDBD-RFX1, pcDNA3.1-RFX1 plasmid with deletion of the sequences encoding the DNA binding domain. **P<0.01. ***P<0.001.

Deletion of RFX1 binding site revealed markedly up-regulation of *Spata4* promoter activity, indicating that RFX1 may function as a significant transcription repressor of *Spata4*. The RFX1 binding sites in human, rat and mouse *Spata4* promoters are highly conserved, and have high similarity with the consensus and other published RFX binding sequences (Figure 3A). Chromatin immunoprecipitation assays using two different RFX1 antibodies showed that RFX1 binds to the mouse *Spata4* promoter in cultured cells. In addition, the sequences containing the GAPDH promoter are not precipitated by RFX1 antibodies (Figure 3B). These results indicated that the RFX1 binding site in the *Spata4* promoter is functional.

Then, we constructed a mutant of mouse RFX1 protein which lacks the DNA-binding domain to further confirm the functional binding of RFX1 to the *Spata4* promoter. Deletion of the DNA-binding domain abolished the regulation of *Spata4* promoter activity by RFX1, demonstrating that the DNA-binding domain is essential for the binding of RFX1 to the *Spata4* promoter (Figure 3C).

### RFX1 regulates endogenous *Spata4* expression in TM4 Sertoli cells

**Figure 4:**
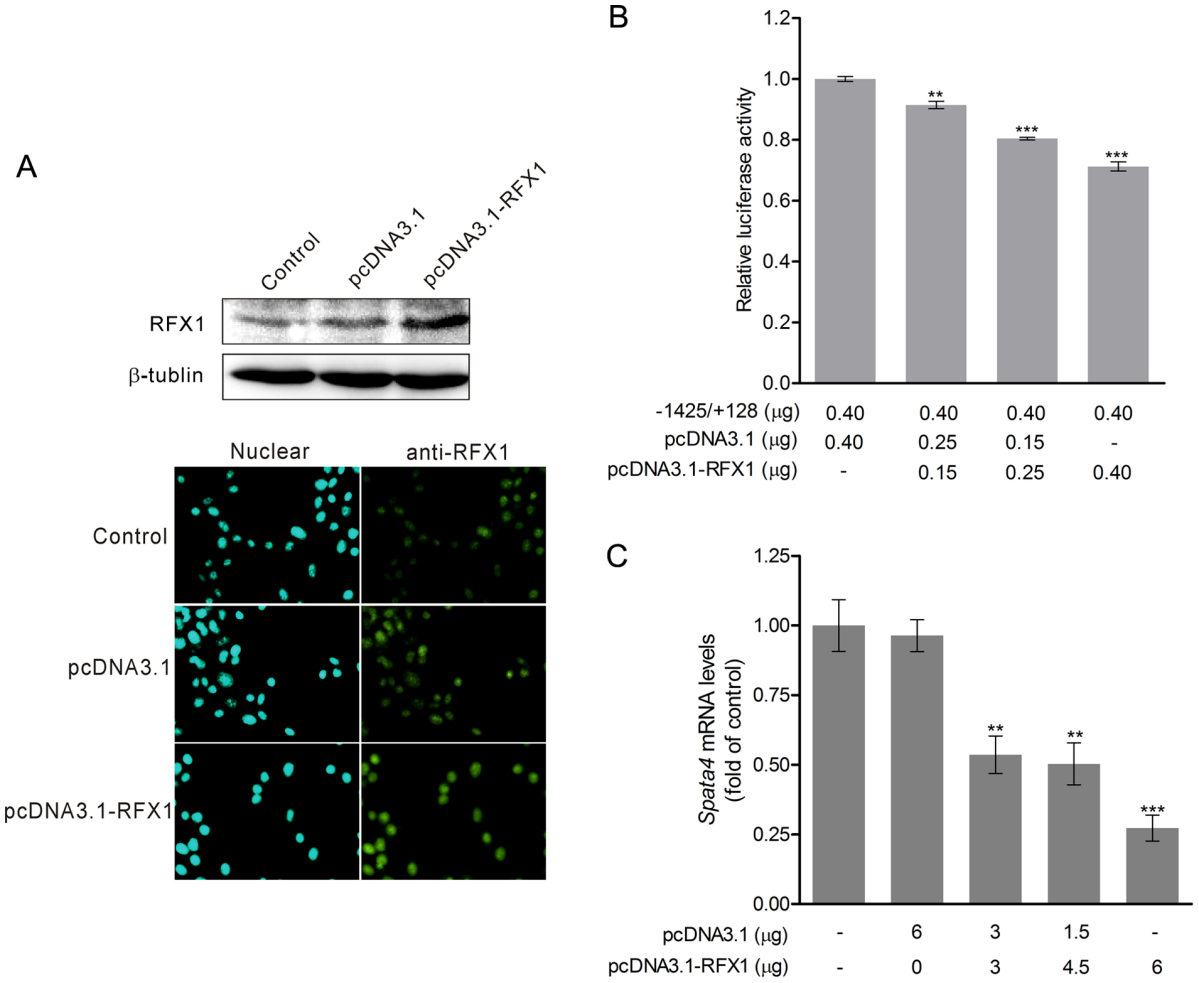
RFX1 regulates *Spata4* mRNA expression in mouse Sertoli cells. After transient transfection of pcDNA3.1-RFX1 for 36 h, the overexpressed RFX1 protein was detected by immunocytochemistry staining and Western blot (**A**), overexpression of RFX1 was shown to down-regulate the promoter activity (**B**) and *Spata4* mRNA expression (**C**) and in a dose-dependent manner. The *Spata4* mRNA expression was analyzed by quantitative PCR and normalized to GAPDH. **P<0.01. ***P<0.001.

Spata4 is localized in the cytoplasm of TM4 cells (Figure S1 A). The mRNA level of *Spata4* is dramatically increased on postnatal days 15 in mouse testes (Figure S1 B). Furthermore, the expression of *Spata4* is abundant in primary Sertoli cells harvested from mouse testis (Figure S1 C). To investigate whether RFX1 regulates endogenous *Spata4* expression, we transiently overexpressed different amounts of RFX1 in TM4 cells and detected its effect on the promoter activity and the mRNA levels of *Spata4*. Overexpression of RFX1 was confirmed by western blotting and immuno-fluorescent assay (Figure 4A). Not only the promoter activity but also the mRNA level of *Spata4* was significantly decreased by RFX1 in a dose dependent manner (Figure 4 B and C).

**Figure 5:**
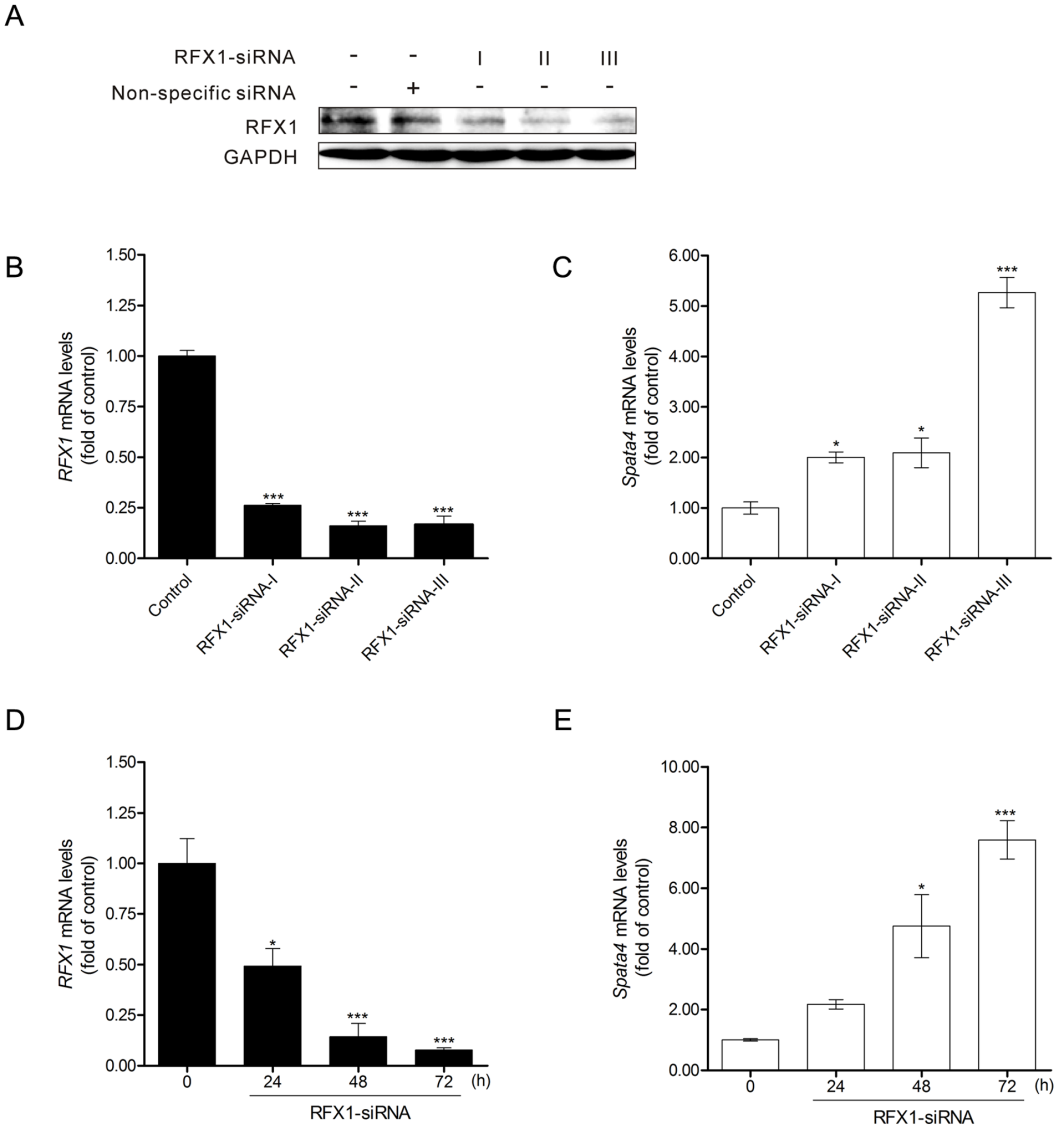
Knockdown of RFX1 could up-regulate endogenous *Spata4* expression. Treatment of mouse RFX1-RNAi-I, -II and -III for 48 h could efficiently knock down the endogenous RFX1 expression (**A, B**). (**C**) *Spata4* mRNA expression level was elevated upon RFX1 knockdown. Control, cells treated with nonspecific siRNA. (**D**) *RFX1* mRNA expression level was decreased by RFX1-RNAi-III treatment. (**E**) *Spata4* mRNA expression level was significantly elevated by RFX1-RNAi-III treatment in a time-dependent manner. *P<0.05. ***P<0.001.

Next, RFX1 expression was knocked down by RNA interfere in TM4 cells (Figure 5 A and B). The mRNA level of *Spata4* was significantly up-regulated by RFX1 siRNA interference (Figure 5C). In addition, endogenous *Spata4* gene expression could be markedly increased by RFX1 knockdown in a time-dependent manner (Figure 5 D and E).

### RFX1 may affect proliferation of Sertoli cells through regulating *Spata4* expression

**Figure 6:**
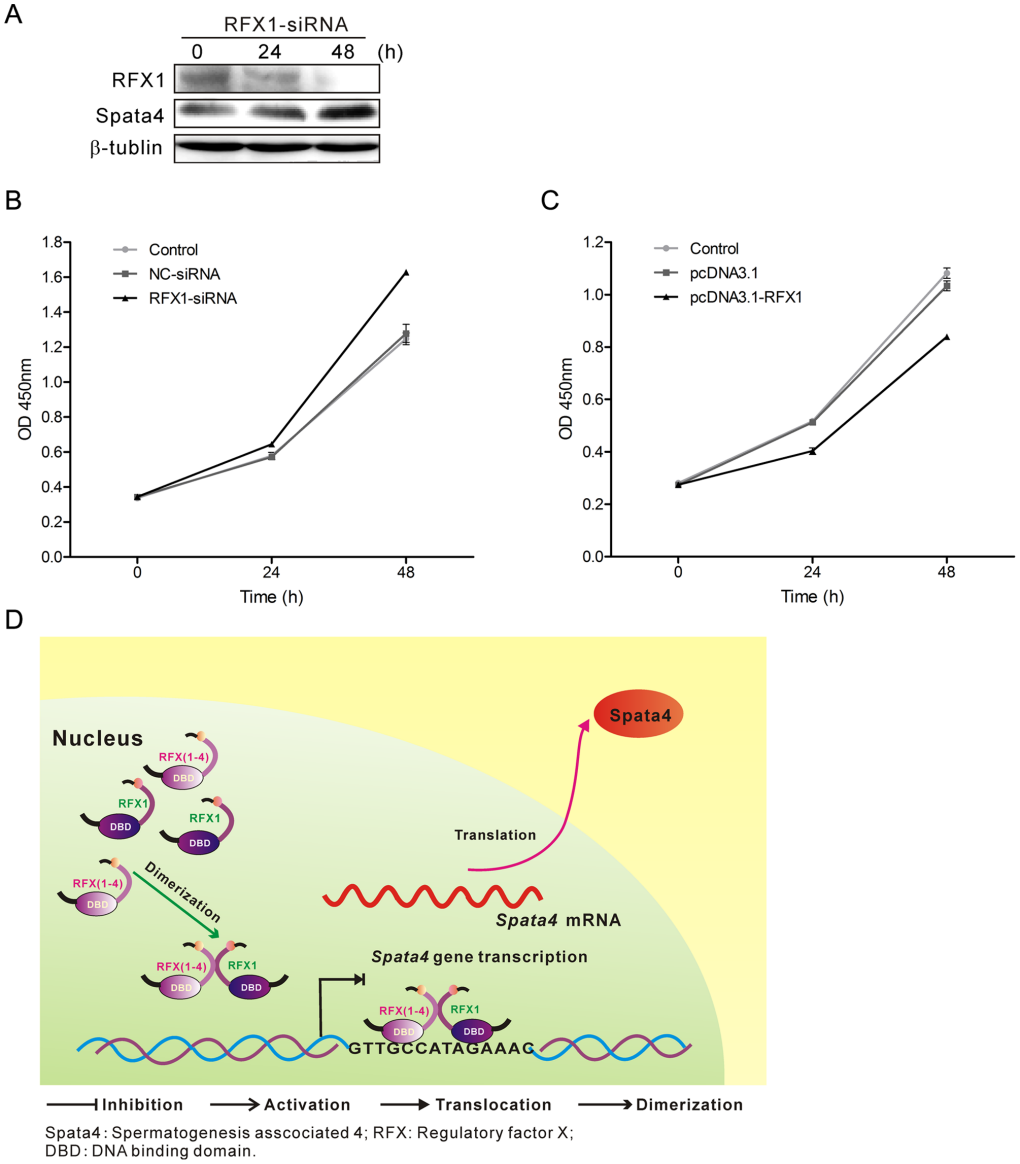
RFX1 is involved in Spata4 mediated cell growth. (**A**) The protein level of RFX1 was decreased while Spata4 was increased in a time-dependent manner with RFX1-siRNA transfection in TM4 cells. (**B**) RFX1 was transiently overexpressed in TM4 cells for 0, 24 and 48 h, (**C**) TM4 cells were treated with Nonspecific-siRNA (50 nM) and RFX1-siRNA (50 nM) for 0, 24 and 48 h, then cell growth was measured by CCK-8 assay. Data are shown as means ± S.E.M., *P*<0.05. (**D**) The regulation of Spata4 by RFX1 and their roles in Sertoli cell proliferation are illustrated. RFX1 dimerizes in the nucleus, then the dimer binds to the concensus 14-bp elements in *Spata4* gene promoter region via the DBD domain and inhibits *Spata4* gene transcription. Down-regulated Spata4 then decreases the growth rate of Seroli cells.

Cell proliferation is closely linked to cell cycling, and cell cycle arrest can lead to suppression of proliferation rate. Exogenous expression of Spata4 in MCF7 cells significantly accelerates cell growth by traversing the S-phase and entering the G2-phase [8]. Meanwhile, stable overexpression of Spata4 could also promote Sertoli cell cycling; thereby improve Sertoli cell proliferation (Figure S2 A). Knockdown of RFX1 increased the expression of Spata4 protein in TM4 cells and the cell growth rate (Figure 6 A and C, Figure S2 B), while overexpression of RFX1 could decrease the cell growth rate (Figure 6B, Figure S2 B), suggesting that RFX1 affects the proliferation of Sertoli cells *in vitro* and that the inhibitory effect was consistent with its regulatory effect on Spata4. The regulation of Spata4 by RFX1 and their roles in Sertoli cell proliferation are illustrated in Figure 6D. Taken together, these results indicated that endogenous expression of Spata4 is important for Sertoli cell growth.

## Discussion

Spata4, conserved in male reproductive system, is important in several biological processes, including cell growth, proliferation and differentiation. However, the transcriptional regulation of *Spata4* was not thoroughly investigated. Here we identified the promoter region of *Spata4* and studied the mechanism of its transcriptional regulation. The mouse *Spata4* promoter contains a CpG island and possesses dispersed transcription start sites, whereas no core promoter elements, such as the TATA box, Initiator (Inr) and Downstream Promoter Element (DPE) were found around the transcription start sites of *Spata4*.

Bioinformatics prediction revealed that the *Spata4* promoter contains potential binding sites of several transcription factors, including AP1, SP1, RFX1, RREB1 and HSF1. Luciferase reporter assays showed that the region between −131 and +128 of the *Spata4* promoter accounts for its basal transcriptional activity. SP1, which binds to the consensus sequence of CCCGCCC that exists in many CpG island with high GC content, may involve in the transcription initiation of the CpG island promoters [34]. Deletion of the SP1 consensus binding sites located between −131, −32 markedly decreased the promoter activity, suggesting that SP1 may function as an important factor in recruiting transcription apparatus to *Spata4* promoter and regulating the transcription activity of *Spata4*.

RFX1, a dual function transcription regulator, has both transcriptional activation and repression properties depending on the promoter context [18]. Mutation of the RFX1 binding site increased the transcriptional activity of the *Spata4* promoter significantly. Furthermore, overexpression of RFX1 reduced both the *Spata4* promoter activity and the mRNA levels of *Spata4*, while knockdown of RFX1 had the opposite effects. Consequently, RFX1 was a remarkable negative regulator of *Spata4* expression.

The RFX proteins bind to DNA as dimers and RFX1-4 can form either heterodimers or homodimers [17], [35]–[37], resulting in the RFX complex possesses similar specificities of binding sites. RFX1 is ubiquitously detected in various tissues with little variability [17]. RFX2 was the most prominent RFX mRNA detected in mouse testis. RFX3 and RFX4 were prevalent in testis [17], yet the RFX4 splice variants in testis are defective due to lack of the transcriptional activation region or the DNA-binding domain [35], [38]. RFX1, RFX2 and RFX3, share high similarity and have identical binding site specificities and the ability of cross-dimerization. The interaction among RFX1, RFX2 and RFX3 and their roles in regulation of *Spata4* promoter activity are therefore worthy of further investigation. Of particular interest, it remains to be uncovered whether other testis specific co-activators are required for RFX1 or other RFX proteins.

In conclusion, we identified the transcription factor RFX1 as a novel negative regulator of *Spata4* gene promoter activity, and provided insight into the regulation of *Spata4* gene. Given the specificity of gene expression pattern of *Spata4* in testis, identification of such a transcription factor brings us closer to the understanding of Spata4 dependent processes including spermatogenesis and cryptorchidism development, and also helps to uncover more biological functions of Spata4.

## Materials and Methods

### Ethics statement

The Animal Protocol (AP) was approved by the Institutional Animal Care and Use Committee of Tsinghua University (IACUCTHU) (AP#: 11-WZ1) with animal care performed strictly according to established institutional guidelines. All surgery was performed under pentobarbital anesthesia, and all efforts were made to minimize suffering.

### Plasmid constructs

The mouse genomic sequence of the 5′-flanking region of the *Spata4* gene including the transcription start site was obtained from GenBank (accession no. NC_000074). DNA extracted from the whole blood cells from C57/BL6 mice with TIANamp Blood DNA Kit (TIANGEN Biotech., Beijing, China) was used as the template. Different truncations of mouse *Spata4* promoter were cloned into the Kpn I and Xho I restriction sites of the pGL4.17 vector (Promega, Madison, WI). The expression plasmid for wild-type RFX1 and was constructed by cloning the corresponding sequence into Kpn I and Xho I restriction sites of pCDNA3.1 myc-His A vector (Invitrogen, Carlsbad, CA). Mutation PCR reactions were performed with PrimSTAR HS polymerase (Takara Bio Inc., Shiga, Japan), 5′-end of the purified PCR products was kinated and ligated using DNA Kination Kit (Takara) referring to the manufacturer's protocol. The primers used during the plasmid construction are shown in Table S1 and S2.

### Cell culture

Mouse Sertoli cell TM4 were cultured in DMEM/F-12 (Thermo Scientific HyClone, Logan, UT), supplemented with 10% fetal bovine serum (Thermo Scientific HyClone), 100 U/ml penicillin and 100 μg/ml streptomycin, at 37°C in a humidified atmosphere containing 5% CO_2_.

### 5′ Rapid amplification cDNA ends

Rapid Amplification of cDNA Ends (RACE) was performed using the SMART^TM^ RECE cDNA Amplification kit (Clontech, Shiga, Japan) following manufacturers' protocols, to determine the transcription start sites of mouse Spata4. Ploy A+ RNA was purified from testis total RNA of 2-months-old C57 BL/6J mice by Oligotex^TM^ mRNA Purification system (Qiagen Inc., Valencia, CA). 5′-RACE-Ready cDNA was synthesized using 5′-CDS (Clontech) primer and SMART II A oligo (Clontech). 5′ RACE PCR was performed with 5′-RACE UPM (Clontech) and *Spata4* specific primer: 5′-GCAGGACAGAACGACTCAGGCGGG-3′, followed by a nested PCR using 5′- nested UPM (Clontech) and a nested *Spata4*-specific primer: 5′- TCCTGCGGGAACTGCTGGTGGTGTAG-3′. As a negative control, 5′-RACE Ready cDNA was amplified with 5′-RACE UPM only. The nested PCR products were then sub-cloned into pMD19-T vector (Takara) and a total of 60 positive clones were sequenced.

### Bioinformatics analysis

Mulan (Multiple-sequence local alignment) analysis was carried out comparing the genomic sequences of about 2000 bp upstream *Spata4* first exon of human, mouse and rat, combined multiTF (http://multitf.dcode.org/) scanning for conserved transcription factor binding sites (TFBS).

### Cell transfection and luciferase assay

TM4 cells (1×10^5^ cells/ml) were seeded in 24-well plates in antibiotic-free culture medium the day before transfection. Each well of cells were transiently transfected with 0.8 μg of *Spata4* reporter plasmids and 0.08 μg *Renilla* reporter plasmid (phRL-TK, Promega) as an internal control using Lipofectamine^TM^ 2000 (Invitrogen). Alternatively, cells were co-transfected with 0.4 μg *Spata4* reporter plasmids, 0.04 μg phRL-TK plasmid and 0.4 μg total amounts of pcDNA3.1 and RFX1 expressing plasmids in a dose dependent manner. Cells were incubated with the DNA-Lipofectamine^TM^ 2000 complex for 6 h, then washed gently with phosphate-buffered saline (PBS) buffer, and cultured in fresh serum-supplemented media for an additional 18 h. Cell lysates were collected and used for measurement of the relative promoter activity of each fragment with the dual luciferase reporter assay system (Promega). pGL4.17 [*luc2*] vector contains no promoter region was used as a control.

### RNAi knockdown experiment

Small interfering RNA knockdown experiments were performed with synthesized siRNA (Genechem, Shanghai, China). Target sequences of RFX1 for siRNA are shown as follow: RFX1-siRNA-I, 5′-AGAACACTGCACAGATCAA-3′; RFX1-siRNA-II, 5′-ACTGTGACAATGTGCTGTA-3′; RFX1-siRNA-III, 5′-TCATGGTAAACCTGCAGTT-3′. Non-specific siRNA was synthesized as forward: 5′- UUCUCCGAACGUGUCACGUtt-3′, reverse: 5′-ACGUGACACGUUCGGAGAAtt-3′. TM4 cells were used in RNAi knockdown experiments. Cells were transfected with the small interfering RNA using Lipofectamine RNAiMAX transfection reagent (Invitrogen) according to the manufacturer's instructions. The three different siRNA (I, II, and III) against RFX1 were tested, and RFX1-siRNA-III were used for further RNAi knockdown experiments.

### Chromatin Immunoprecipitation (ChIP) Assay

ChIP was performed using the Magnetic Chromatin Immunoprecipitation Kit (Active Motif, Carlsbad, CA) as described in the manufacturer's protocol. Briefly, TM4 cells were washed once with phosphate buffered saline and then fixed in culture medium containing 1% formaldehyde at room temperature for 10 min. Cells were then collected and lysed to release the nucleus. The nucleus was digested with the enzyme mix at 37°C for 10 min to shear the chromatin into small segments. 2 μg of RFX1 (I-19) antibodies (Santa Cruz Biotechnology, Santa Cruz, CA), 2 μg of RFX1 (D-19) antibodies (Santa Cruz), or a negative control IgG (Abcam, Cambridge, UK) were used for immunoprecipitation of the sheared chromatin at 4°C for 4 h. The pulled-down chromatin was washed, reverse cross-linked and purified. PCR primers for mouse *Spata4* promoter were forward: 5′-CACAGAGCCTTCTTGGAGG-3′ and reverse: 5′-GGGAACTGCTGGTGGTGTA-3′. PCR primers for mouse *GAPDH* promoter were forward: 5′-GCCCTTGAGCTAGGACTGGAT-3′ and reverse: 5′-CCTGGCACTGCACAAGAAGAT-3′.

### Quantitative real-time polymerase chain reaction (RT-qPCR)

Total RNA was extracted from TM4 cells using Trizol reagent (Invitrogen) according to the manufacturer's protocol. cDNA was transcribed with RevaTra Ace reverse transcriptase (Toyobo, Osaka, Japan) using oligo (dT)_20_ as primers. Each cDNA transcribed from 0.8 μg RNA was subsequently used for RT-qPCR analysis with SYBR Green Real-time PCR Mater Mix (Toyobo) in CFX96 Real-Time PCR Detection Systems (Bio-Rad, Hercules, CA). PCR primers for mouse *Spata4* were forward: 5′-CAGATACAAGTCAAGAGGTTC-3′ and reverse: 5′-GTGTTCTCACAAGGATTTCCAC -3′. PCR primers for mouse *RFX1* were forward: 5′-TGCTGCCCCTGCACCCTCACA-3′ and reverse: 5′-GGTGGGAACACCGGTCTGGCTT-3′. PCR primers for mouse *GAPDH* were forward: 5′-CATGGCCTTCCGTGTTCCTA-3′ and reverse: 5′-CCTGCTTCACCACCTTCTTGAT-3′. All the primers are intron-spanning. The specificity of primers was confirmed by the melting curves which performed on the amplified products. The thermal cycling conditions were as follows: initial denaturation at 95°C for 10 min, followed by 40 cycles of denaturing at 95°C for 15 s, annealing at 60°C for 30 s and extension at 72°C for 30 s.

### Western blotting

Cells were collected and lysed with the cell lysis buffer (Beyotime, Shanghai, China). Alternatively, nuclear protein was isolated with the Nuclear and Cytoplasmic Protein Extraction Kit (Beyotime). Protein concentrations were determined using the BCA Protein Assay Kit (Thermo Scientific). Protein samples were separated by electrophoresis through SDS-PAGE gels and transferred to polyvinylidene difluoride membranes (Millipore, Schwalbach, Germany). The membranes were blocked at room temperature for 1.5 h in Tris-buffered saline containing 0.05% Tween 20 with 5% non-fat dry milk. The membranes were then incubated with primary antibodies specific for RFX1 (1∶500; Santa Cruz), Spata4 (1∶1000; prepared by our laboratory), GAPDH (1∶1000; Cwbiotech, Beijing, China) or β-tublin (1∶1000; Cwbiotech) at 4°C overnight, followed by an incubation with horseradish peroxidase-conjugated anti-goat IgG (1∶5000; Santa Cruz) or anti-rabbit IgG (1∶10000) at room temperature for 1 h. The chemiluminescence reaction was performed using ECL reagent (Thermo Scientific).

### Immunofluorescence staining

TM4 cells were seeded on a 6-well dish with a density of 1×10^5^ cells/ml and fixed with methylate-acetone (1∶1) for 30 min. After blocking with 10% bovine serum albumin (BSA) for 1 hour, a primary antibody against RFX1 (1∶100; Santa Cruz) was added for 2 h, followed by an anti-goat IgG conjugated with FITC (1∶100; Santa Cruz) for 1 h. DAPI (0.1 mg/ml) was added to label the nucleus for 10 min. A negative control without the primary antibody was used to monitor the staining background. Images were obtained through Leica DX100 (Leica Microsystems Wetzlar GmbH, Germany).

### CCK-8 assay

TM4 cells were suspended at a final concentration of 2×10^4^ cells/ml and seeded in 96-well plates, then transiently transfected with pcDNA3.1-vector, pcDNA3.1-RFX1, non-specific siRNA, siRNA-RFX1, respectively. After incubation for the indicated time as 0, 24 and 48 h, cell growth was determined by the CCK-8 assay (Beyotime). In brief, 10 μl CCK-8 solutions were added to each well and cultures were incubated at 37°C for 2 h. The absorbance at 450 nm was measured with a Benchmark microplate reader (Bio-Rad).

### Statistical analysis

The values reported in graphs are the means ± S.E.M. from three independent experiments. One-way ANOVA followed by Bonferroni multiple comparison was performed to assess the differences. A value of *P*<0.05 was considered statistically significant.

## Supporting Information

Figure S1 **Spata4 protein localization in Sertoli cells and its expression in mouse testis.** (A) Spata4 protein localization in TM4 cells via immunofluorescence. Fluorescein isothiocyanate (FITC) staining of Spata4 (green; anti-Spata4) is shown. Propidium iodide (PI) staining of cell nuclei (red), a composite image (Merge) and a negative control (Neg) are presented. Images shown were captured at 630 fold magnification via confocal laser scanning microscopy. (B) RT-qPCR analysis of mouse Spata4 expression in developing testis including day 7, day 11, day 15, day 21, day 30, day 60. As shown in the figure, expression of mouse *Spata4* can be detected after the mouse is 15 days old. (C) Expression of Spata4 in mouse Sertoli cells. The mRNA levels of *Spata4* in primary Sertoli cells, TM4 Sertoli cells and testes are determined by RT-PCR. Columns and bars indicate the mean ± S.E.M. of values independently repeated three times.
(TIF)

Figure S2 **Effect of Spata4 on cell cycle distribution and cell proliferation of TM4 Sertoli cells.** (A) Confluent TM4 and TM4/Spata4 cells (1×10^7^ cells/10 cm dish) were harvested and cell cycle distribution was determined by flow cytometry analysis. (B) RFX1 was transiently overexpressed in TM4 cells for 0, 24 and 48 h, and TM4 cells were treated with Nonspecific-siRNA (50 nM) and RFX1-siRNA (50 nM) for 0, 24 and 48 h, then total cell number were counted. Data are shown as means ± S.E.M., *P*<0.05.
(TIF)

Table S1 **PCR primers used for constructing the reporter plasmids of the mouse Spata4 promoter.**
(DOC)

Table S2 **PCR primers used for constructing the wild-type and mutant mouse RFX1.**
(DOC)

## References

[1] Liu SF, He S, Liu BW, Zhao Y, Wang Z (2004) Cloning and characterization of testis-specific spermatogenesis associated gene homologous to human SPATA4 in rat. Biol Pharm Bull 27: 1867–1870. 15516739 10.1248/bpb.27.1867

[2] Weitzel JM, Shiryaeva NB, Middendorff R, Balvers M, Radtke C, et al (2003) Testis-specific expression of rat mitochondrial glycerol–3-phosphate dehydrogenase in haploid male germ cells. Biol Reprod 68: 699–707. 12533437 10.1095/biolreprod.102.008540

[3] Olesen C, Larsen NJ, Byskov AG, Harboe TL, Tommerup N (2001) Human FATE is a novel X-linked gene expressed in fetal and adult testis. Mol Cell Endocrinol 184: 25–32. 11694338 10.1016/s0303-7207(01)00666-9

[4] Ogi T, Mimura J, Hikida M, Fujimoto H, Fujii-Kuriyama Y, et al (2001) Expression of human and mouse genes encoding polkappa: testis-specific developmental regulation and AhR-dependent inducible transcription. Genes Cells 6: 943–953. 11733032 10.1046/j.1365-2443.2001.00478.x

[5] Imai-Senga Y, Sun-Wada GH, Wada Y, Futai M (2002) A human gene, ATP6E1, encoding a testis-specific isoform of H (+)-ATPase subunit E. Gene. 289: 7–12. 10.1016/s0378-1119(02)00542-5 12036578

[6] Liu SF, Ai C, Ge ZQ, Liu HL, Liu BW, et al (2005) Molecular cloning and bioinformatic analysis of SPATA4 gene. J Biochem Mol Biol 38: 739–747. 16336790 10.5483/bmbrep.2005.38.6.739

[7] Wang X, Harimoto K, Liu J, Guo J, Hinshaw S, et al (2011) Spata4 promotes osteoblast differentiation through Erk-activated Runx2 pathway. J Bone Miner Res 26: 1964–1973. 21445983 10.1002/jbmr.394

[8] Liu SF, Lu GX, Liu G, Xing XW, Li LY, et al (2004) Cloning of a full-length cDNA of human testis-specific spermatogenic cell apoptosis inhibitor TSARG2 as a candidate oncogene. Biochem Biophys Res Commun 319: 32–40. 15158438 10.1016/j.bbrc.2004.04.160

[9] Griswold MD (1998) The central role of Sertoli cells in spermatogenesis. Semin Cell Dev Biol 9: 411–416. 9813187 10.1006/scdb.1998.0203

[10] Eddy EM (2002) Male germ cell gene expression. Recent Prog Horm Res 57: 103–128. 12017539 10.1210/rp.57.1.103

[11] Tanaka H, Baba T (2005) Gene expression in spermiogenesis. Cell Mol Life Sci 62: 344–354. 15723169 10.1007/s00018-004-4394-y PMC11924428

[12] Orth JM, Gunsalus GL, Lamperti AA (1988) Evidence from Sertoli cell-depleted rats indicates that spermatid number in adults depends on numbers of Sertoli cells produced during perinatal development. Endocrinology 122: 787–794. 3125042 10.1210/endo-122-3-787

[13] Antequera F, Bird A (1993) Number of CpG islands and genes in human and mouse. Proc Natl Acad Sci U S A 90: 11995–11999. 7505451 10.1073/pnas.90.24.11995 PMC48112

[14] Gardiner-Garden M, Frommer M (1987) CpG islands in vertebrate genomes. J Mol Biol 196: 261–282. 3656447 10.1016/0022-2836(87)90689-9

[15] Aftab S, Semenec L, Chu JS, Chen N (2008) Identification and characterization of novel human tissue-specific RFX transcription factors. BMC Evol Biol 8: 226. 18673564 10.1186/1471-2148-8-226 PMC2533330

[16] Emery P, Durand B, Mach B, Reith W (1996) RFX proteins, a novel family of DNA binding proteins conserved in the eukaryotic kingdom. Nucleic Acids Res 24: 803–807. 8600444 10.1093/nar/24.5.803 PMC145730

[17] Reith W, Ucla C, Barras E, Gaud A, Durand B, et al (1994) RFX1, a transactivator of hepatitis B virus enhancer I, belongs to a novel family of homodimeric and heterodimeric DNA-binding proteins. Mol Cell Biol 14: 1230–1244. 8289803 10.1128/mcb.14.2.1230-1244.1994 PMC358479

[18] Katan Y, Agami R, Shaul Y (1997) The transcriptional activation and repression domains of RFX1, a context-dependent regulator, can mutually neutralize their activities. Nucleic Acids Res 25: 3621–3628. 9278482 10.1093/nar/25.18.3621 PMC146931

[19] Tsang SY, Nakanishi M, Peterlin BM (1990) Mutational analysis of the DRA promoter: cis-acting sequences and trans-acting factors. Mol Cell Biol 10: 711–719. 2105459 10.1128/mcb.10.2.711-719.1990 PMC360870

[20] Kouskoff V, Mantovani RM, Candeias SM, Dorn A, Staub A, et al (1991) NF-X, a transcription factor implicated in MHC class II gene regulation. J Immunol 146: 3197–3204. 2016543

[21] Reinhold W, Emens L, Itkes A, Blake M, Ichinose I, et al (1995) The myc intron-binding polypeptide associates with RFX1 in vivo and binds to the major histocompatibility complex class II promoter region, to the hepatitis B virus enhancer, and to regulatory regions of several distinct viral genes. Mol Cell Biol 15: 3041–3048. 7760800 10.1128/mcb.15.6.3041 PMC230535

[22] Chen L, Smith L, Johnson MR, Wang K, Diasio RB, et al (2000) Activation of protein kinase C induces nuclear translocation of RFX1 and down-regulates c-myc via an intron 1 X box in undifferentiated leukemia HL-60 cells. J Biol Chem 275: 32227–32233. 10918054 10.1074/jbc.M002645200

[23] Sengupta PK, Ehrlich M, Smith BD (1999) A methylation-responsive MDBP/RFX site is in the first exon of the collagen alpha2(I) promoter. J Biol Chem 274: 36649–36655. 10593968 10.1074/jbc.274.51.36649

[24] Sengupta P, Xu Y, Wang L, Widom R, Smith BD (2005) Collagen alpha1(I) gene (COL1A1) is repressed by RFX family. J Biol Chem 280: 21004–21014. 15788405 10.1074/jbc.M413191200 PMC1382295

[25] Liu M, Lee BH, Mathews MB (1999) Involvement of RFX1 protein in the regulation of the human proliferating cell nuclear antigen promoter. J Biol Chem 274: 15433–15439. 10336433 10.1074/jbc.274.22.15433

[26] Wang KR, Nemoto T, Yokota Y (2007) RFX1 mediates the serum-induced immediate early response of Id2 gene expression. J Biol Chem 282: 26167–26177. 17630394 10.1074/jbc.M703448200

[27] Alam KY, Frostholm A, Hackshaw KV, Evans JE, Rotter A, et al (1996) Characterization of the 1B promoter of fibroblast growth factor 1 and its expression in the adult and developing mouse brain. J Biol Chem 271: 30263–30271. 8939980 10.1074/jbc.271.47.30263

[28] Ray SK, Yang XQ, Chiu IM (1997) Transcriptional activation of fibroblast growth factor 1.B promoter is mediated through an 18-base pair cis-acting element. J Biol Chem 272: 7546–7555. 9054460 10.1074/jbc.272.11.7546

[29] Hsu YC, Liao WC, Kao CY, Chiu IM (2010) Regulation of FGF1 gene promoter through transcription factor RFX1. J Biol Chem 285: 13885–13895. 20189986 10.1074/jbc.M109.081463 PMC2859551

[30] Kistler WS, Horvath GC, Dasgupta A, Kistler MK (2009) Differential expression of Rfx1-4 during mouse spermatogenesis. Gene Expr Patterns 9: 515–519. 19596083 10.1016/j.gep.2009.07.004 PMC2761754

[31] Carninci P, Sandelin A, Lenhard B, Katayama S, Shimokawa K, et al (2006) Genome-wide analysis of mammalian promoter architecture and evolution. Nat Genet 38: 626–635. 16645617 10.1038/ng1789

[32] Loots GG, Ovcharenko I (2007) Mulan: multiple-sequence alignment to predict functional elements in genomic sequences. Methods Mol Biol 395: 237–254. 17993678 PMC3704129

[33] Ovcharenko I, Loots GG, Giardine BM, Hou M, Ma J, et al (2005) Mulan: multiple-sequence local alignment and visualization for studying function and evolution. Genome Res 15: 184–194. 15590941 10.1101/gr.3007205 PMC540288

[34] Antequera F (2003) Structure, function and evolution of CpG island promoters. Cell Mol Life Sci 60: 1647–1658. 14504655 10.1007/s00018-003-3088-6 PMC11138798

[35] Morotomi-Yano K, Yano K, Saito H, Sun Z, Iwama A, et al (2002) Human regulatory factor X 4 (RFX4) is a testis-specific dimeric DNA-binding protein that cooperates with other human RFX members. J Biol Chem 277: 836–842. 11682486 10.1074/jbc.M108638200

[36] Gajiwala KS, Chen H, Cornille F, Roques BP, Reith W, et al (2000) Structure of the winged-helix protein hRFX1 reveals a new mode of DNA binding. Nature 403: 916–921. 10706293 10.1038/35002634

[37] Reith W, Herrero-Sanchez C, Kobr M, Silacci P, Berte C, et al (1990) MHC class II regulatory factor RFX has a novel DNA-binding domain and a functionally independent dimerization domain. Genes Dev 4: 1528–1540. 2253877 10.1101/gad.4.9.1528

[38] Blackshear PJ, Graves JP, Stumpo DJ, Cobos I, Rubenstein JL, et al (2003) Graded phenotypic response to partial and complete deficiency of a brain-specific transcript variant of the winged helix transcription factor RFX4. Development 130: 4539–4552. 12925582 10.1242/dev.00661

