# Supplementary figures and images for: Spermatogenesis Associated 4 Promotes Sertoli Cell Proliferation Modulated Negatively by Regulatory Factor X1
Source: PLoS One. 2013 Oct 11;8(10):e75933. doi: 10.1371/journal.pone.0075933 (PMC3795713; doi:10.1371/journal.pone.0075933)

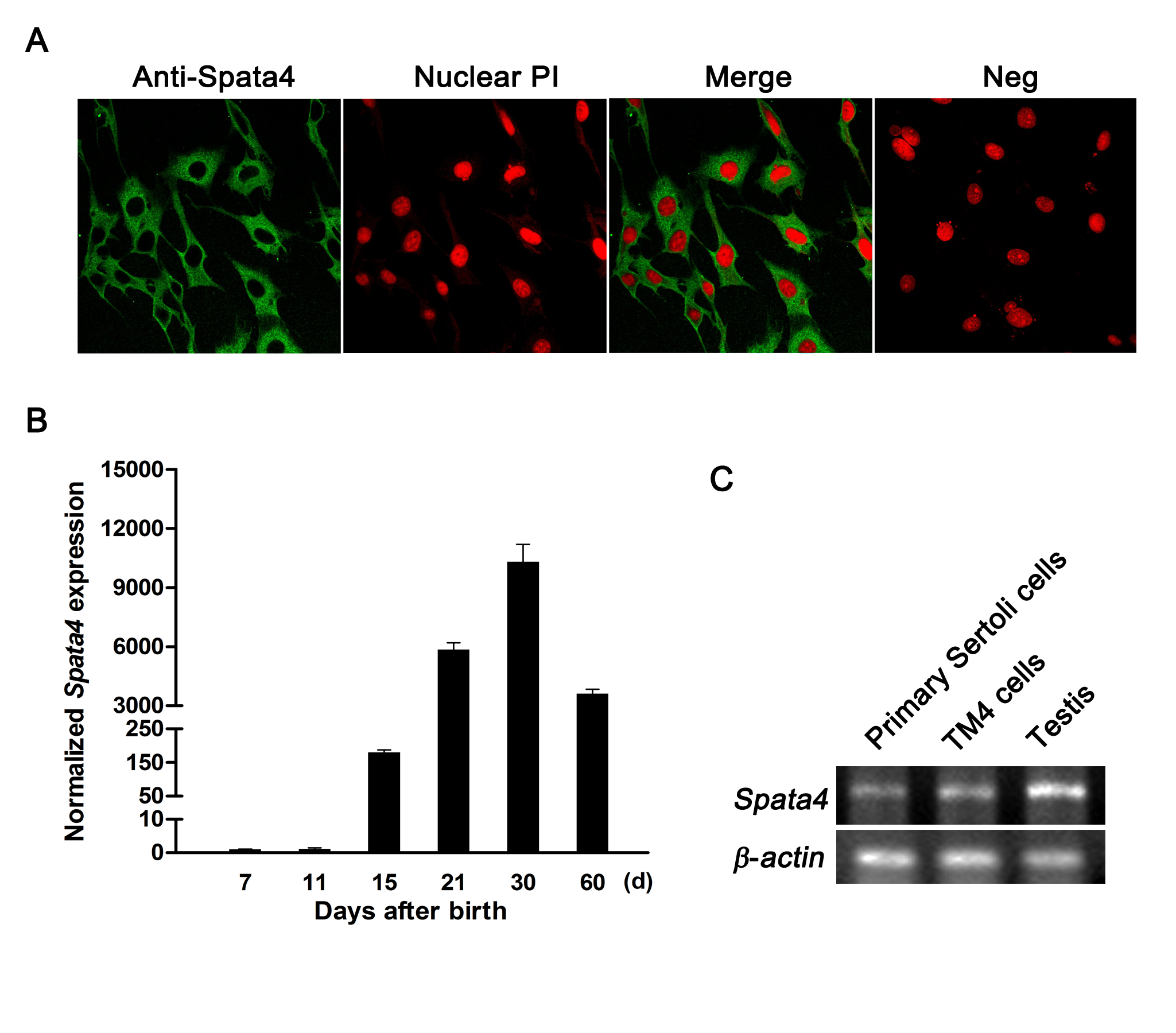

Supplement: Figure S1 — Spata4 protein localization in Sertoli cells and its expression in mouse testis. (A) Spata4 protein localization in TM4 cells via immunofluorescence. Fluorescein isothiocyanate (FITC) staining of Spata4 (green; anti-Spata4) is shown. Propidium iodide (PI) staining of cell nuclei (red), a composite image (Merge) and a negative control (Neg) are presented. Images shown were captured at 630 fold magnification via confocal laser scanning microscopy. (B) RT-qPCR analysis of mouse Spata4 expression in developing testis including day 7, day 11, day 15, day 21, day 30, day 60. As shown in the figure, expression of mouse Spata4 can be detected after the mouse is 15 days old. (C) Expression of Spata4 in mouse Sertoli cells. The mRNA levels of Spata4 in primary Sertoli cells, TM4 Sertoli cells and testes are determined by RT-PCR. Columns and bars indicate the mean ± S.E.M. of values independently repeated three times. (TIF) [file pone.0075933.s001.tif]

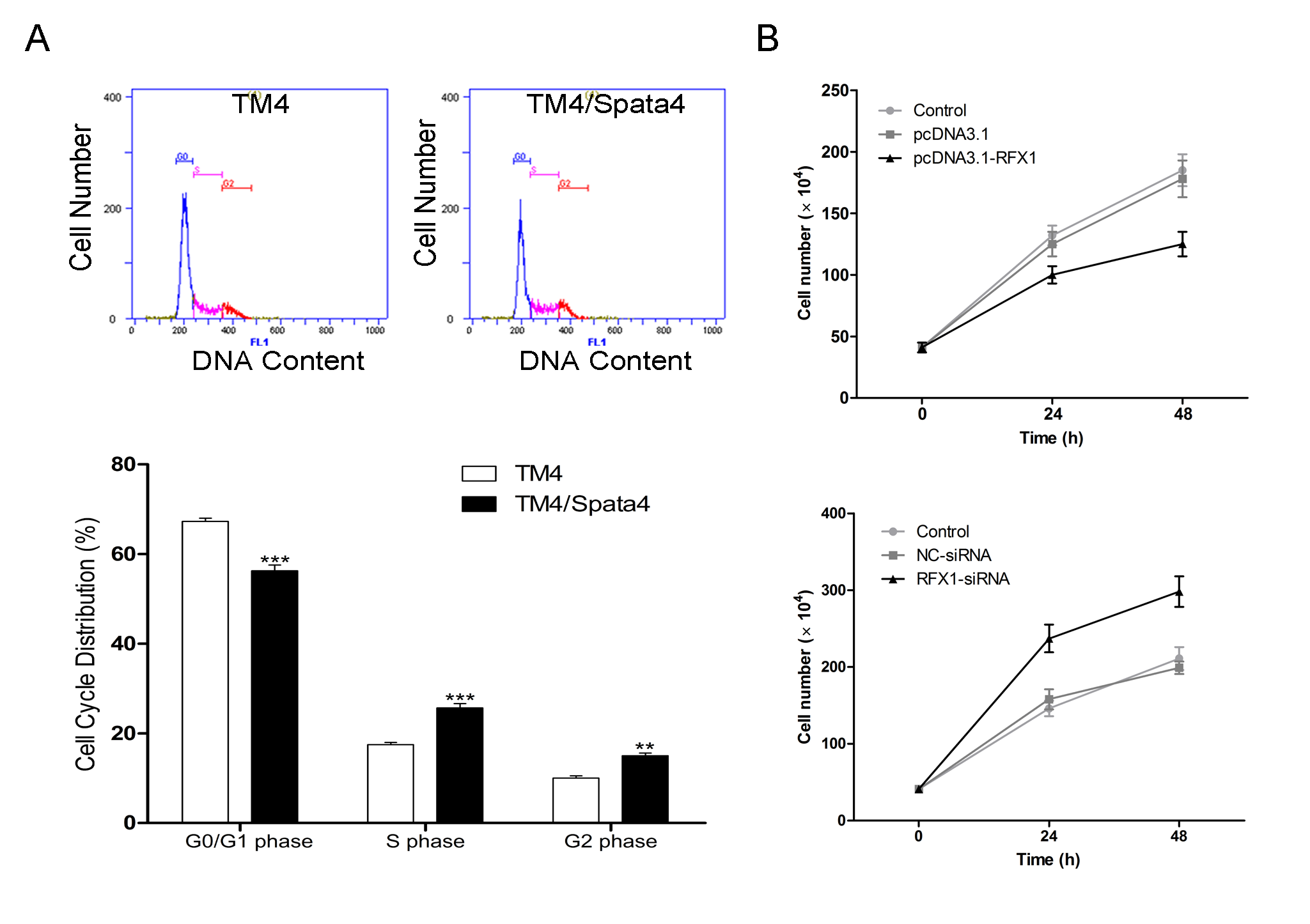

Supplement: Figure S2 — Effect of Spata4 on cell cycle distribution and cell proliferation of TM4 Sertoli cells. (A) Confluent TM4 and TM4/Spata4 cells (1×10^7^ cells/10 cm dish) were harvested and cell cycle distribution was determined by flow cytometry analysis. (B) RFX1 was transiently overexpressed in TM4 cells for 0, 24 and 48 h, and TM4 cells were treated with Nonspecific-siRNA (50 nM) and RFX1-siRNA (50 nM) for 0, 24 and 48 h, then total cell number were counted. Data are shown as means ± S.E.M., P<0.05. (TIF) [file pone.0075933.s002.tif]
