# Supplementary material for: Spermatogenesis Associated 4 Promotes Sertoli Cell Proliferation Modulated Negatively by Regulatory Factor X1
Source: PLoS One. 2013 Oct 11;8(10):e75933. doi: 10.1371/journal.pone.0075933 (PMC3795713; doi:10.1371/journal.pone.0075933)
Supplement: Table S1 — PCR primers used for constructing the reporter plasmids of the mouse Spata4 promoter. (DOC) [file pone.0075933.s003.doc]

Table S1 PCR primers used for constructing the reporter plasmids of the mouse Spata4 promoter

| **Primer^a^** | **Sequence (5' to 3')^b^** |
| --- | --- |
| Forward primers |  |
| F-2819 | TAATTCACCAGTTCAGGGAG |
| F-2339 | ATACTTTTGGAGGAAGGACG |
| F-1824 | GAACTGCTTTGTGGAGTTAC |
| F-1425 | TCCTAAATCTTGGTTGGTGG |
| F-904 | TAGTAGCAAGCGCCTTAATC |
| F-373 | CTACTCTCATCCACCTC |
| F-131 | CGTTCATGGATCCAGAG |
| F-32  F-del-RFX1  F-del-SP1  F-del-HSF1  F-del-RREB1  F-del-AP1 | ATCGAGGCTTTAGCGTC  ATAAAGCACCATGGCTGCCGCCGGCC  TTCGACGCTAAAGCCTCGATTCCAG  ACAGTGAAAATTGTTTTTTTTTTTT  ACAATT TTCACTGTCTTCTAGAGC  ATAAGACATATATCACTGGACAAAGAG |
| Reverse primers |  |
| R+128  R-del-RFX1  R-del-SP1  R-del-HSF1  R-del-RREB1  R-del-AP1 | CGTCTTGGATGATTCTGC  ATCAGCTGGGGGCTGATAAGGTCTGC  CTTCGGCCTCCAAGAAGGCTCTGTG  CATTTCTCTTCAAAATTCTTTATTGAAC  GGGGGGAGGTGGATGAGAGTAGGAGATA  TAATGACTGTTTTTTAAGAAACTGTC |

^a^The number indicates the position of the 5' terminus of the primer relative to the first transcription start site of mouse Spata4.

^b^The primer sequences do not include the restriction sites and the protection bases.
