# Supplementary material for: Spermatogenesis Associated 4 Promotes Sertoli Cell Proliferation Modulated Negatively by Regulatory Factor X1
Source: PLoS One. 2013 Oct 11;8(10):e75933. doi: 10.1371/journal.pone.0075933 (PMC3795713; doi:10.1371/journal.pone.0075933)
Supplement: Table S2 — PCR primers used for constructing the wild-type and mutant mouse RFX1. (DOC) [file pone.0075933.s004.doc]

Table S2 PCR primers used for constructing the wild-type and mutant mouse RFX1

| **Primer** | **Sequence (5' to 3')** |
| --- | --- |
| Forward primers |  |
| F-mus-RFX1^a^ | GAATGGCAACACAGTCCTATGTT |
| F-△DBD-RFX1 | TACCACTATTACGGCCTGCGGATCAAAG |
| Reverse primers |  |
| R-mus-RFX1^a^ | TTAGCTGGAGGGCAGGGCCT |
| R-△DBD-RFX1 | GGAGTAAGACTGGCTGGCATTGCCTAGC |

^a^The primer sequences do not include the restriction sites and the protection bases.
